# Adequacy of care management of patients with polyhandicap in the French health system: A study of 782 patients

**DOI:** 10.1371/journal.pone.0199986

**Published:** 2018-07-06

**Authors:** Marie-Christine Rousseau, Thierry Billette de Villemeur, Sherezad Khaldi-Cherif, Catherine Brisse, Agnès Felce, Karine Baumstarck, Pascal Auquier, on behalf of the French Polyhandicap Group

**Affiliations:** 1 Fédération des Hôpitaux de Polyhandicap et Multihandicap Hôpital San Salvadour, Assistance Publique Hôpitaux de Paris, Hyères, France; 2 EA 3279, CEReSS—Health Service Research and Quality of Life Center, Aix Marseille Université, 27 bd Jean Moulin, Marseille, France; 3 Sorbonne Université, UPMC, GRC ConCer-LD and AP-HP, Hôpital Trousseau, Service de Neuropédiatrie—Pathologie du développement, Paris, France; 4 Centre de référence des déficits intellectuels de causes rares, Inserm, Paris, France; 5 Hôpital de La Roche Guyon, Service de Polyhandicap Pédiatrique, Assistance Publique Hôpitaux de Paris, La Roche Guyon, France; 6 Union Générale Caisse Assurance Maladie (UGECAM), Ile de France, Paris, France; 7 Comité d'Études, d'Éducation et de Soins Auprès des Personnes Polyhandicapées, Paris, France; 8 Hôpital d’Hendaye, Assistance Publique Hôpitaux de Paris, Hendaye, France; Centre Hospitalier Universitaire Vaudois, FRANCE

## Abstract

**Background:**

The aims of this study were 1) to describe the health profiles and care management of polyhandicapped patients according to 2 modalities, specialized rehabilitation centers (SRC) and residential facilities (RF), and 2) to estimate the adequacy of care management of these patients.

**Methods:**

This was an 18-month cross-sectional study including patients with a combination of severe motor deficiency and profound intellectual impairment. The patients were from 4 SRC and 9 RF. The following data were collected: sociodemographics, health status, care management, and adequacy of care management.

**Results:**

A total of 782 patients were included: 410 (52%) were cared for in SRC and 372 (48%) in RF. Global objective adequacy (health severity and age category) was higher for patients cared for in SRC compared with patients cared for in RF (57 vs. 44%, p< = 10^−3^). Global subjective adequacy (self-perception of the referring physician and request of change in structure) was higher for patients cared for in SRC in comparison with patients cared for in RF (98 vs. 92%, p< = 10^−3^).

**Conclusions:**

This study provides key elements of adequacy of care management modalities for polyhandicapped patients in France.

**Trial registration:**

ClinicalTrials.gov NCT02400528

## Introduction

Polyhandicap is a complex disability condition corresponding to a chronic affliction occurring in an immature brain, leading to a combination of profound intellectual disability and serious motor deficit, resulting in extreme restriction of autonomy and communication. This definition was adopted by the French scientific community and by French law (French Law n° 89–798, 1989, October 27th, health policy of care disability). Polyhandicap is close to the notion of profound intellectual and multiple disabilities used in other countries that does not systematically refer to a disorder affecting an immature brain. Patients with polyhandicap present varying severity of disorders and comorbidities, need permanent health and educational support, are dependent on human and technical assistance [1]. In France, the prevalence is estimated in the pediatric population to be between 0.7 and 1.28 per thousand, i.e., 880 new cases of children with polyhandicap per year [2,3].

The French health system allows these patients to benefit from three main care management modalities: specialized rehabilitation centers (SRC), residential facilities (RF), and home care (HC) [4]. The SRC offer a high level of medical and paramedical physical rehabilitation, a lower level of psychosocial education, and a high level of prevention care for inpatients for a theoretical limited duration. The RF offer a high level of psychosocial education and a lower level of medical care. For these two modalities, units are dedicated for adult and pediatric populations. HC corresponds to patients (adults and children) living at home; the family may benefit from specific nursing and medical care for the patient. No robust data are available on the number of patients cared for at home.

This healthcare pathway is meant to optimize the care management of patients with polyhandicap according to their specific needs, in terms of age, dependency degree and health severity. However, concerns have been reported by patients’ families and by health care professionals that some patients seem to be inappropriately and sub-optimally managed [5]. Some patients needing a higher level of medical and paramedical rehabilitation are managed in RF, while some patients needing a rather high level of psychosocial education are managed in SRC. Some families of patients cared for at home are waiting for availability in RF or SRC, for a long time. Some children are hospitalized in adult units and vice versa. This inadequacy could have consequential impacts on the health and well-being of patients and families and on the optimization of health expenditures. Description of patients’ profiles within the different types of existing structures should provide key elements to optimize the global care management of these individuals. To our knowledge, no previous study has provided information about the adequacy of care management for French polyhandicapped patients.

Our objectives are 1) to describe, from a large sample of polyhandicapped patients, the health profiles (handicaps, co-morbidities, autonomy, and neurodevelopmental status) and care management according to 2 modalities (specialized rehabilitation centers and residential facilities); and 2) to estimate the adequacy of care management of these patients.

## Methods

### Design and settings

This cross-sectional study included patients from March 2015 to September 2016. The recruitment of patients cared for in SRC was performed in the following 4 French centers spread over different French territories: San Salvadour Hospital, La Roche Guyon Hospital, Union Générale des Caisses d’Assurance Maladie d’Ile de France (UGECAM-IDF) Hospital, and Hendaye Hospital. These 4 centers include 500 beds (300 dedicated for adults and 200 dedicated for children) and offer predominantly medical care. The patients cared for in RF were recruited from 9 of the 17 centers of the Comité d'Études, d'Education et de Soins Auprès des Personnes Polyhandicapées Association (CESAP). These 17 structures include 730 beds (250 dedicated for adults and 480 for children) and offer predominantly psychoeducational care.

### Inclusion criteria and data collection

**Fig 1:**
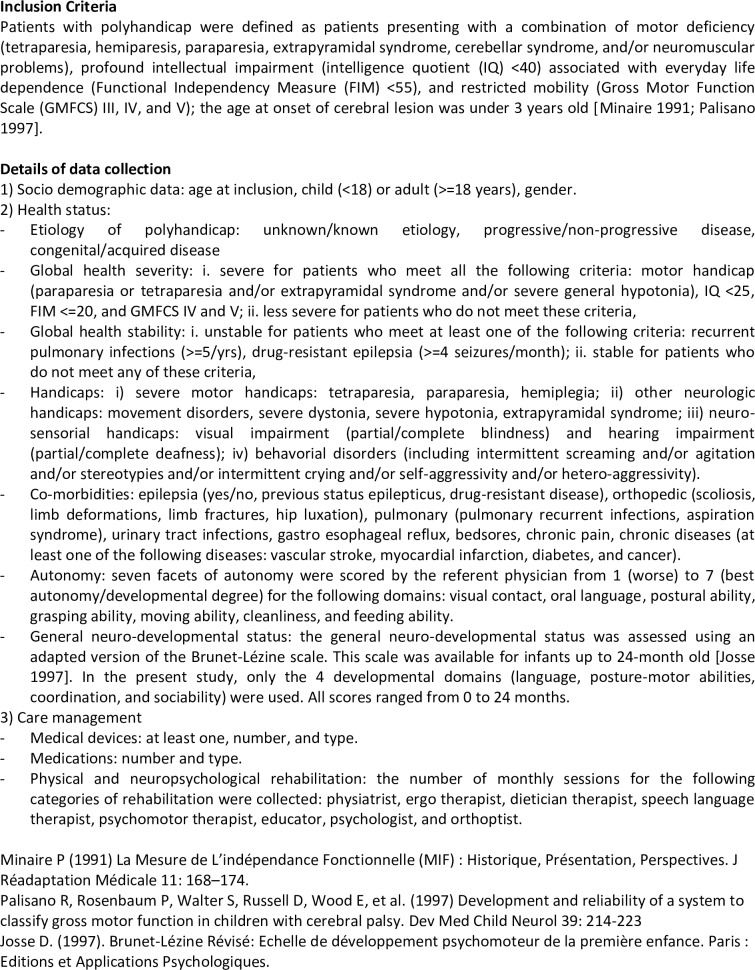
Details of inclusion criteria and data collection.

The main inclusion criteria are presented in Fig 1. The data included the following items: sociodemographic data, health status, and care management and are detailed in Fig 1.

The estimation of adequacy of the care management was defined by the following 2 ways: objective adequacy and subjective adequacy.

1. Objective adequacy was defined from the following 2 indicators:
  - Global health severity adequacy: Adequacy was defined by a patient with a severe global health status who was cared for in an SRC or by a patient with less severe global health status who was cared for in RF.
  - Age adequacy: Adequacy was defined by an individual under 18 years old who was cared for in a unit dedicated to children or by an individual over 18 years old who was cared for in a unit dedicated to adults.
  - Global objective adequacy: Adequacy was defined by an individual who was in adequacy for global health severity and in adequacy for age (defined above). Other cases were defined as inadequacy.
2. Subjective adequacy was defined from the following 3 indicators:
  - Perception of the referring physician (a referring physician is designated for each patient) of medical care adequacy: For each included patient, the referring physician was asked to complete his/her perception of adequacy (adequate vs. not adequate) of the care structure in terms of medical care.
  - Perception of the referring physician of educational care adequacy: For each included patient, the referring physician was asked to complete his/her perception of adequacy (adequate vs. not adequate) of the care structure in terms of educational care.
  - Request for a change of care structure: The request for a change in care structure was collected through two questions from the referring physician: 1. ‘Is the patient registered on a waiting list for another care structure? Yes/no’, and 2. ‘Is there an official request of change of structure made by the Department for Persons with Disabilities? Yes/no’ (the French Department for Persons with Disabilities is in charge of supporting handicapped persons and their relatives). Adequacy was defined by negative answers for the 2 questions.
  - Global subjective adequacy: Adequacy was defined by adequacy for the self-perception of the referring physician in terms of medical care and adequacy for one of the other 2 parameters.

### Ethics

Regulatory monitoring was performed in accordance with the French law that requires the approval of the French ethics committee (Comité de Protection des Personnes Sud Méditerranée V, 20/10/2014, reference number 2014-A00953-44). A written consent form was collected for each participant. Clinical trial number: NCT02400528.

### Statistics

The quantitative data are expressed as the means and standard deviations (SD) or the medians and interquartile ranges (IQR), and the qualitative data are expressed as numbers and percentages. The normality of quantitative parameters was estimated by means of Shapiro-Wilk tests. All the parameters were compared among the 2 groups using Student’s t tests or Mann-Whitney tests for continuous variables, and chi-square tests or Fisher exact tests for qualitative variables. Adequacy proportions were compared among the 2 groups. Logistic regressions were performed to consider adjustment of age (the goodness of fit for the models was tested using the Hosmer–Lemeshow test). The results were presented as odds ratios (OR) and 95% confidence intervals (CI). Concordance between objective and subjective adequacy was determined using kappa coefficients. No data imputation was performed. The statistical analyses were performed using SPSS software (IBM SPSS PASW Statistics Inc., Chicago, IL, USA). All of tests were two-sided. The threshold for statistical significance was set at p <0.05.

## Results

### Sample and sociodemographics

During the 18-month study period, 782 patients were included: 410 (47%, rate inclusion of 95%) cared for in SRC and 372 (43%, rate inclusion of 95%) cared for in RF. The patients cared for in SRC were significantly older and consequently were more often adult patients than patients cared for in RF (8-year difference, p< = 10^−3^). The details are in Table 1.

**Table 1:** General characteristics and health status of polyhandicapped patients according to the care management modality.

		Spec. rehab. centers	Residential facilities		
		N = 410	N = 372		
		N (%)	N (%)	MD%	p
**1. Sociodemographics**					
Age	M±SD	30.5±17.7	22.2±14.1	0	< = 10^−3^ ^+^
Age categories	Children	133 (32)	180 (48)	0	< = 10^−3^
	Adults	277 (68)	192 (52)		
Gender	Boys/Men	218 (53)	197 (53)	0	0.952
	Girls/Women	192 (47)	175 (47)		
**2. Etiology of polyhandicap**					
Known		354 (88)	308 (83)	1.4	0.045
Unknown		47 (12)	62 (17)		
Progressive		192 (49)	26 (7)	2.2	< = 10^−3^
Non progressive		203 (51)	344 (93)		
Acquired		112 (28)	55 (15)	1.7	< = 10^−3^
Congenital		286 (72)	316 (85)		
**3. Global health status**					
Global health severity ^#^	Less severe	165 (41)	177 (48)	1.0	0.058
	Severe	238 (59)	194 (52)		
Global health stability ^@^	Stable	308 (77)	279 (76)	2.0	0.608
	Unstable	90 (23)	89 (24)		
**4. Handicaps**					
Severe motor handicaps	Tetraplegia	311 (80)	236 (74)	10.0	< = 10^−3^
	Paraplegia	74 (19)	61 (19)		
	Hemiplegia	2 (1)	20 (6)		
Neurologic handicaps	Movement disorders	43 (11)	46 (13)	4.1	0.411
	Severe dystonia	31 (8)	34 (10)	5.2	0.471
	Severe hypotonia	36 (9)	153 (42)	4.1	< = 10^−3^
	Extrapyramidal syndrom	113 (29)	64 (18)	4.7	0.001
Sensorial disorders	Visual impairment	121 (30)	98 (27)	2.2	0.367
	Hearing impairment	23 (6)	21 (6)	3.7	0.965
Behaviorial disorders ^^^		377 (94)	224 (61)	1.4	< = 10^−3^
**5. Comorbidities**					
Epilepsia	Presence of epilepsia	225 (56)	203 (56)	1.7	0.919
	Previous status epilepticus	65 (17)	101 (42)	20.7	< = 10^−3^
	Drug-resistant epilepsia	59 (15)	75 (20)	1.4	0.036
Orthopedic	Scoliosis	258 (65)	187 (55)	5.8	0.003
	Limb deformation	314 (78)	240 (67)	2.9	< = 10^−3^
	Limb fracture	24 (6)	28 (8)	4.1	0.415
	Hip luxation	94 (24)	107 (31)	5.2	0.016
Pulmonary	Recurrent infections	54 (14)	24 (7)	1.9	0.001
	Aspiration syndrome	125 (31)	60 (16)	2.2	< = 10^−3^
Urinary tract infections		58 (15)	18 (5)	1.7	< = 10^−3^
Gastroesophageal reflux		195 (49)	121 (33)	2.9	< = 10^−3^
Bedsores		20 (5)	19 (5)	1.5	0.932
Chronic pain		132 (33)	16 (4)	0.9	< = 10^−3^
Chronic diseases *		10 (3)	10 (3)	2.3	0.831

MD: missing data; p: p-value; M±SD: mean ± standard deviation; Med (IQR): median (interquartile range)

^+^ Mann-Whitney test

^#^ Severe case: association of motor handicap, IQ <25, FIM< = 20, and GMFCS IV/V

^@^ Unstable case: recurrent pulmonary infections and/or drug resistant epilepsy

^^^ Behaviorial disorders: intermittent scream 64%, agitation 62%, stereotypies 32%, intermittent crying 50%, self-aggressivity 15%, and hetero-aggressivity 8%

* Chronic diseases: vascular stroke and/or myocardial infarction and/or diabetes and/or cancer

### Health status

The unknown etiologies of polyhandicap were less frequent in patients cared for in SRC than patients cared for in RF (12 vs. 17%, p = 0.045). Patients cared for in SRC had more often a progressive (49%) and acquired (28%) disease than the patients cared for in RF (7 and 15%, p< = 10^−3^). Patients in SRC did not differ from patients cared for in RF for global severity status (59 vs. 52%, p = 0.058). All details are in Table 1 and S1 Table.

### Care management

A higher proportion of patients in SRC needed at least one medical device (48%) in comparison with patients cared for in RF (48 vs. 23%, p< = 10^−3^). The number of daily medications was lower for patients in RF in comparison with patients in SRC (1.7 point difference, p< = 10^−3^). All details are in Table 2 and S2 Table.

**Table 2:** Care management according to the care management modality.

			Spec. rehab. centers	Residential facilities		
			N = 410	N = 372		
			N (%)	N (%)	MD%	p
Medical devices	At least one *		195 (48)	84 (23)	1.2	< = 10^−3^
	Number *	M±SD Med (IQR)	1.2±0.5 1 (1–1)	1.1±0.2 1 (1–1)	1.2	0.011^+^
Medications	Number	M±SD Med (IQR)	8.3±3.3 8 (6–10)	6.6±3.4 6 (4–8)	2.6	< = 10^−3^

MD: missing data; p: p-value; M±SD: mean ± standard deviation; Med (IQR): median (interquartile range)

* including invasive mechanical ventilation, non-invasive mechanical ventilation, tracheotomia, naso gastric tube, gastrostomy, permanent urinary probe, cerebrospinal fluid derivation, central venous catheter; ^+^ Mann-Whitney test

### Adequacy of care management

**Fig 2:**
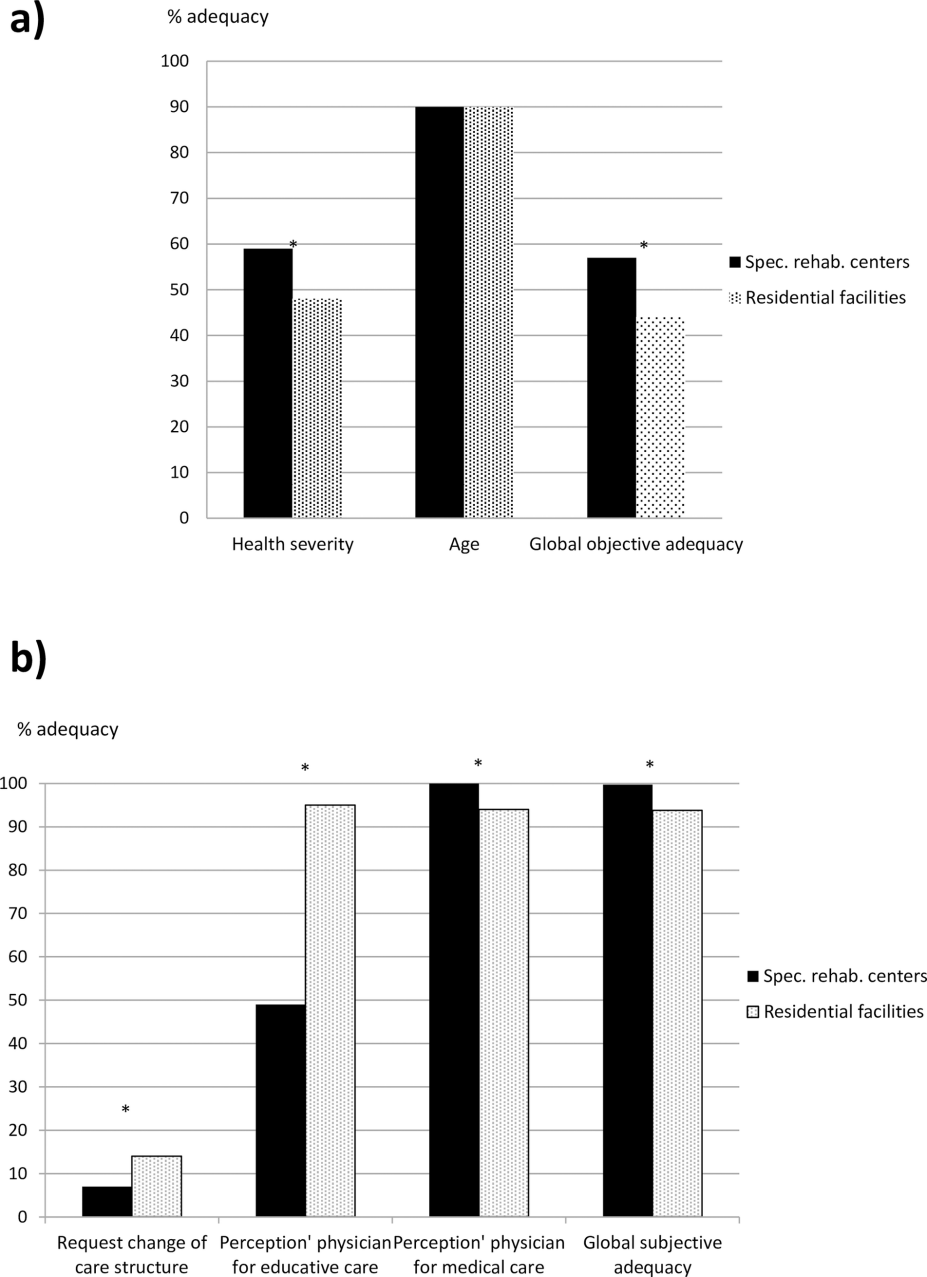
Adequacy of care management. a) Objective adequacy of care management. b) Subjective adequacy of care management.

The proportion of patients in SRC in an adequate care structure regarding health severity (proportion of severe cases, 59%) was higher than for the individuals of RF (proportion of less severe cases, 48%, p< = 10^−3^). Adequacy regarding age of the patient (90%) did not differ between the 2 groups. Global objective adequacy was higher for patients cared for in SRC compared with patients cared for in RF (57 vs. 44%, p< = 10^−3^). The results are presented in Fig 2A. After adjusting for the main confounding factors (age, global health severity, global health instability, and medical devices), the objective adequacy was still better for patients cared for in SRC (OR = 1.6, IC = 1.1–2.1, p = 0.007).

The referring physicians of patients considered that 1) medical care was more adapted for patients cared for in SRC in comparison with patients cared for in RF (100 vs. 94%, p< = 10^−3^); and 2) psychoeducational care was more adapted for patients cared for in RF in comparison with patients cared for in SRC (95 vs. 49%, p< = 10^−3^). A change of structure was requested for fewer patients cared for in SRC in comparison with patients cared for in RF (7 vs. 14%, p<0.004). Global subjective adequacy was higher for patients cared for in SRC (98%) in comparison with patients cared for in RF (98 vs. 92%, p< = 10^−3^). The results are presented in Fig 2B. After adjusting for the main confounding factors (age, global health severity, global health instability, and medical devices), the subjective adequacy was still better for patients cared for in SRC in comparison with patients cared for in RF (OR = 4.9, IC = 1.8–13.7, p = 0.002).

In the subgroup including the patients cared for in SRC and the patients cared for in RF, there was no concordance between global objective adequacy and global subjective adequacy (kappa coefficient 0.06, p< = 10^−3^).

## Discussion

To date, in France, no robust data allow for one to have a faithful picture of health profiles and care management of patients with polyhandicap. From an observational design, the present study provides a substantial and innovative description of patients with polyhandicap in terms of the adequacy of care management.

The adequacy of care management should be analyzed from the perspective of the health status severity of the patients. Because SRC were supposed to provide high-level (technical and specialized) medical care for patients presenting with the most severe health status for a limited duration, while RF were supposed to offer more educational care for less severe individuals, we hypothesized that patients in SRC would probably present with a more severe clinical disease than the patients cared for in RF. This assumption was partially confirmed by our findings. Although the severity of the health of the patients in SRC, based on our definition, was more altered than that of the patients in RF, two questions should be addressed. Were the 41% of patients with less severe health status cared for in SRC managed in the most appropriate care structure with regard to their health status and medical/educational needs? Conversely, did the 52% of patients with severe health status cared for in RF receive adequate medical care? Some answers should be suggested. First, the definition of the severity that we proposed may be inappropriate. Second, the patients in SRC still presented specific health indicators that may exclude or prohibit care management in RF or at home, including high rate of comorbidities and consequently more medications and medical devices, low autonomy and neuropsychomotor developmental level, high proportion of unstable health status (recurrent pulmonary infection, resistant epilepsy), and a high rate of behavioral disorders. Authors have previously reported that these disorders remain very difficult to manage by families and were a source of social instability and familial inconvenience [6]. Instability and noisy behavioral disorders may prompt a family to choose a non-familial care structure when the patient is growing and aging.

The adequacy of care management should also be analyzed from the perspective of the population (child or adult population) the structure is devoted to. For patients cared for in SRC, 10% of the patients were not in a structure adapted to his/her age. This result is in accordance with a French survey showing that the proportion of adults with multiple disabilities maintained in children's institutions in France was estimated at 13% (http://fulltext.bdsp.ehesp.fr/Ministere/Drees/EtudesResultats/2016/946/er946.pdf). If health decision-making authorities are to reconsider availability and capacities offered by the different structures, the transition process from adolescence to adulthood should be highlighted. Often under-documented, this transition is a source of reluctance of families and healthcare staff [7]. In polyhandicap, as in other chronic health conditions, a dysfunctional transition process leads to adverse consequences in health [8,9]. Future specific programs should be thought of to improve this process.

Due to its binary nature (adequate or inadequate), this objective adequacy, based on the severity and the age category of the patient, should be questioned and weighted by other subjective aspects. The global subjective adequacy, based on the perceived adequacy of the referring physicians of the patients and on the request of change of care structure, should be examined. The subjective adequacy was better for patients cared for in SRC in comparison with patients cared for in RF. As they considered that medical care was appropriate for patients cared for in SRC, half of the referring physicians considered that the patients partially received inappropriate psychoeducational care. The very long median duration stay (often a lifelong stay) [4] in these SRC should be taken into consideration by health decision-making authorities. Despite a strained economic health system, it should be necessary to develop appropriate psychoeducational care that this population deserves.

Finally, the discrepancy between the adequacy based on objective indicators (44–57%) and the adequacy based on subjective indicators (87–98%) should be questioned. From the healthcare teams’ point of view, the needs in terms of patients’ specific care management should not be limited to age and severity. The clinician's perception takes into account multiple factors such as the patient's needs in terms of prevention of comorbidities and handicaps, the presence of behavioral disorders (90% of patients in SRC), the relative resources in terms of healthcare, and the capacity of caregivers to care for the patient; all of these may sometimes justify the maintenance of some patients in less objectively adequate care management facilities. Future studies should more specifically explore these aspects using mixed approaches (quali-quantitative researches).

The representativeness of our sample should be discussed. We can assume that the 4 SRC included in this present study should provide a high representativeness of patients with polyhandicap cared for in SRC. Approximately 70% of hospital beds dedicated to polyhandicap in France were located in these 4 centers [4]. The representativeness of the other group of RF is more questionable. The recruitment of patients cared for in RF was exclusively based on a voluntary basis. Future studies should consider longitudinal design and medico-economic aspects to complement this cross-sectional study.

## Conclusions

The French health care system offers two main care management organizations for patients with polyhandicap (specialized rehabilitation centers and residential facilities). This observational study showed that the adequacy of care management from objective indicators (health severity and age) seems less satisfactory than the perceived adequacy of the physicians. These preliminary elements should be taken into account by clinicians, caregivers, and health decision-making authorities to optimize the global care management of these patients.

## Supporting information

S1 Checklist Strobe checklist. (DOCX)

S1 Table Autonomy and neurodevelopment status according to the care management modality. (DOCX)

S2 Table Details for medical devices, medications, and rehabilitation according to the care management modality. (DOCX)
