# Supplementary material for: Adequacy of care management of patients with polyhandicap in the French health system: A study of 782 patients
Source: PLoS One. 2018 Jul 6;13(7):e0199986. doi: 10.1371/journal.pone.0199986 (PMC6034799; doi:10.1371/journal.pone.0199986)
Supplement: S1 Table — (DOCX) [file pone.0199986.s002.docx]

**S1 Table. Autonomy and neurodevelopment status according to the care management modality**

|  |  | **Spec. rehab.**  **centers** | **Residential**  **facilities** |  |  |
| --- | --- | --- | --- | --- | --- |
|  |  | **N=410** | **N=372** |  |  |
| **Autonomy** |  | **Med (IQR)** | **Med (IQR)** | **MD%** | **p** |
| Visual contact (1-7) ^a^ |  | 3 (1 - 5) | 4 (2 - 6) | 0.8 | <=10^-3 +^ |
| Oral language (1-7) ^a^ |  | 2 (2 - 2) | 2 (2 - 3) | 1.5 | <=10^-3 +^ |
| Postural ability (1-7) ^a^ |  | 3 (1 - 3) | 3 (2 - 4) | 0.9 | <=10^-3 +^ |
| Grasping ability (1-7) ^a^ |  | 1 (1 – 3) | 2 (1 – 5) | 1.0 | <=10^-3 +^ |
| Moving ability (1-7) ^a^ |  | 1 (1 – 1) | 1 (1 – 3) | 0.5 | <=10^-3 +^ |
| Cleanliness (1-7) ^a^ |  | 1 (1 – 1) | 1 (1 – 1) | 1.7 | 0.004 ^+^ |
| Feeding ability (1-7) ^a^ |  | 3 (1 – 3) | 3 (3 – 3) | 0.6 | <=10^-3 +^ |
| **Neurodevelopment status** |  | **Med (IQR)** | **Med (IQR)** | **MD%** | **p** |
| Language **^b^** |  | 2 (2 - 6) | 5 (3 - 8) | 18.0 | <=10^-3 +^ |
| Posture-motor ability **^b^** |  | 3 (2 - 7) | 5 (2 - 9) | 16.2 | <=10^-3 +^ |
| Coordination **^b^** |  | 3 (2 - 6) | 4 (3 - 7) | 20.2 | 0.001 ^+^ |
| Sociability **^b^** |  | 3 (2 - 6) | 5 (3 - 9) | 17.4 | <=10^-3 +^ |

Med (IQR): median (interquartile range); MD: missing data; p: p-value

^a^ Autonomy scores from 1 (worse) to 7 (highest autonomy/global development)

**^b^** Neurodevelopmental status levels range from 0 to 24 months

^+^ Mann-Whitney test
