# Supplementary material for: Adequacy of care management of patients with polyhandicap in the French health system: A study of 782 patients
Source: PLoS One. 2018 Jul 6;13(7):e0199986. doi: 10.1371/journal.pone.0199986 (PMC6034799; doi:10.1371/journal.pone.0199986)
Supplement: S2 Table — (DOCX) [file pone.0199986.s003.docx]

**S2 Table. Details for medical devices, medications, and rehabilitation according to the care management modality**

|  |  | **Spec. rehab.**  **centers** | **Residential**  **facilities** |  |  |
| --- | --- | --- | --- | --- | --- |
|  |  | **N=410** | **N=372** |  |  |
|  |  | **N (%)** | **N (%)** | **MD%** | **p** |
| Medical devices | Invasive MV | 7 | 0 | 0.9 | 0.016* |
|  | Non-invasive MV | 19 (5) | 0 | 1.2 | <=10^-3^ |
|  | Tracheotomia | 11 (3) | 2 | 1.0 | 0.019 |
|  | Naso gastric tube | 7 | 4 | 1.0 | 0.439 |
|  | Gastrostomy | 171 (42) | 72 (19) | 0.8 | <=10^-3^ |
|  | Permanent urinary probe | 2 | 2 | 0.9 | 1.00* |
|  | CSF derivation | 16 (4) | 8 (2) | 0.8 | 0.149 |
|  | Central venous catheter | 2 | 0 | 0.8 | 0.500* |
| Medications | Laxatives | 330 (92) | 281 (77) | 7.2 | <=10^-3^ |
|  | Antiepileptic | 303 (75) | 275 (75) | 1.5 | 0.836 |
|  | Antalgics | 375 (92) | 109 (30) | 1.0 | <=10^-3^ |
|  | Psychotrops | 250 (72) | 137 (38) | 9.1 | <=10^-3^ |
|  | Osteoporosis prevention | 144 (39) | 136 (37) | 5.9 | 0.544 |
|  | Antispastics | 112 (31) | 84 (23) | 6.1 | 0.019 |
|  | Antidystonics | 79 (22) | 65 (18) | 5.9 | 0.193 |
|  | Martial suplementation | 75 (21) | 42 (12) | 7.9 | <=10^-3^ |
|  |  | **Med (IQR)** | **Med (IQR)** |  |  |
| Rehabilitation ^°^ | Physiatrist | 8 (0 - 20) | 4 (0 - 8) | 8.6 | <=10^-3 +^ |
|  | Ergotherapist | 0 (0 - 0) | 0 (0 - 2) | 14.6 | 0,127 ^+^ |
|  | Dietician therapist | 1 (0 - 1) | 0 (0 - 0) | 25.0 | <=10^-3 +^ |
|  | Speach langage therapist | 0 (0 - 0) | 0 (0 - 0) | 19.6 | 0,220 ^+^ |
|  | Psychomotor therapist | 0 (0 - 4) | 0 (0 - 4) | 16.0 | 0.027 ^+^ |
|  | Educator | 0 (0 - 4) | 8 (0 - 23) | 25.1 | <=10^-3 +^ |

p: p-value; Med (IQR): median (interquartile range); MD: missing data

^*^ Fisher exact’ test; ^+^ Mann-Whitney test; ^°^ Number of monthly sessions
